# Use of Mobile Crowdsensing in Disaster Management: A Systematic Review, Challenges, and Open Issues

**DOI:** 10.3390/s23031699

**Published:** 2023-02-03

**Authors:** Didem Cicek, Burak Kantarci

**Affiliations:** School of Electrical Engineering and Computer Science, University of Ottawa, Ottawa, ON K1N 6N5, Canada

**Keywords:** mobile crowdsensing, mobile crowdsourcing, smartphone sensors, disaster management, emergency management, systematic literature review

## Abstract

With the increasing efforts to utilize information and communication technologies (ICT) in disaster management, the massive amount of heterogeneous data that is generated through ubiquitous sensors paves the way for fast and informed decisions in the case of disasters. Utilization of the big “sensed” data leads to an effective and efficient management of disaster situations so as to prevent human and economic losses. The advancement of built-in sensing technologies in smart mobile devices enables crowdsourcing of sensed data, which is known as mobile crowdsensing (MCS). This systematic literature review investigates the use of mobile crowdsensing in disaster management on the basis of the built-in sensor types in smart mobile devices, disaster management categories, and the disaster management cycle phases (i.e., mitigation, preparedness, response, and recovery activities). Additionally, this work seeks to unveil the frameworks or models that can potentially guide disaster management authorities towards integrating crowd-sensed data with their existing decision-support systems. The vast majority of the existing studies are conceptual as they highlight a challenge in experimental testing of the disaster management solutions in real-life settings, and there is little emphasis on the use cases of crowdsensing through smartphone sensors in disaster incidents. In light of a thorough review, we provide and discuss future directions and open issues for mobile crowdsensing-aided disaster management.

## 1. Introduction

Disasters are unforeseen events that happen naturally or that are human-made, causing significant damage, destruction, and human suffering. Disasters cause tremendous losses, both economic and human, all over the world and disaster response systems have grown in significance particularly after September 11th (2001), the London bombings (2005), and Japan’s tsunami (2011) [1]. To name one out of many examples, Canada has a history of disasters with recently increased occurrence of wildfires and floods triggered by climate change [2]. United Nations countries have adopted the UN Sendai Framework for Disaster Risk Reduction (2015–2030) to enable member countries to set nation-wide visions on disaster management, which is an integrated set of activities to establish the capabilities to prepare for, respond to, recover from, or mitigate against a disaster [3,4].

**Figure 1:**
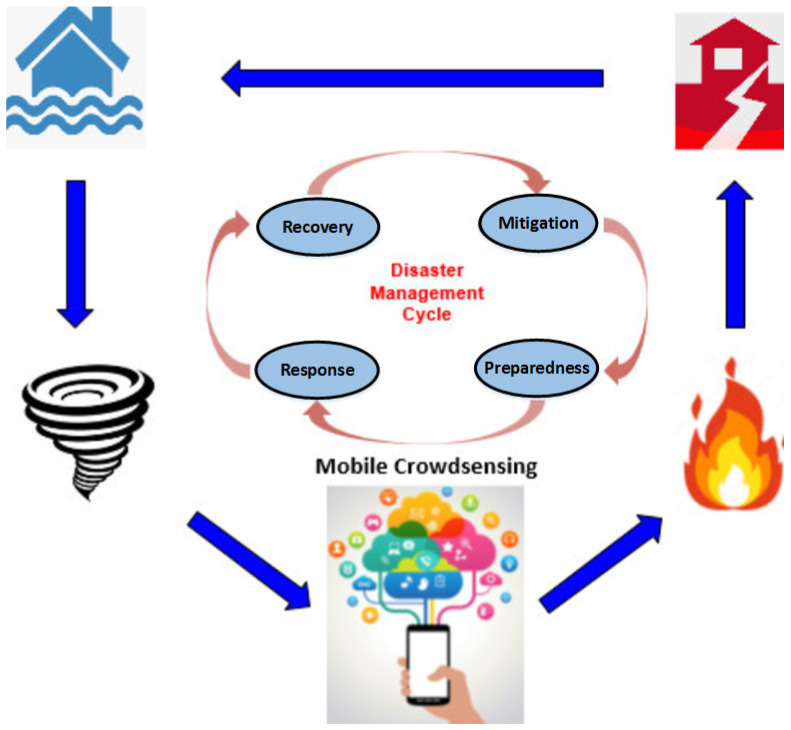
Mobile Crowdsensing-Aided Disaster Management.

It is often challenging to effectively monitor and respond to disasters. It is of paramount importance to rescue authorities to receive and process real-time data quickly in responding to a disaster to prevent potential human and economic losses [5]. Crowdsensing has recently emerged as a new problem-solving method for disasters, opening up a new space to generate disaster responses by improving situational awareness through collecting real-time sensor data and feeding into the disaster management cycle [6]. Figure 1 illustrates potential integration and use of crowdsensing in disaster management. Crowdsensing is a mass data sensing activity leveraging volunteer crowds to collect insights on the environment or on a specific phenomenon of interest through various types of sensors such as smartphone sensors or IoT devices that are equipped with suitable sensing infrastructure [7]. The advancement in sensor technologies and ever-increasing number of sensors built-in smartphones such as GPS, camera, microphone, accelerometer, gyroscope, barometer, pedometer, light, or proximity facilitates a low-cost sensing system [8].

The tipping point of using crowdsourcing in disaster management was the Haiti Earthquake in 2010 where 640 volunteers contributed to building road maps of Haiti to distribute disaster aid in two weeks [9]. Crowdsourcing and Crowdsensing are two different concepts that are sometimes used interchangeably. Crowdsourcing, a model to leverage crowd intelligence through online human input to serve specific organizational goals [10], mostly relies on gathering unstructured crowd intelligence through social media and online social networks. However, crowdsensing refers to a more structured type of data generation by crowds through physical sensors [11]. Mobile crowdsensing that facilitates data generation by crowds through smartphone sensors is the focal.

Crowdsensing, with its capabilities to provide real-time, diverse, and low-cost data, stands out as a recent and promising technology that can offer solutions in disaster management [12]. Few use cases of crowdsensing in disaster management could be stated as detection of hazards, locating community resources or identification of victim evacuation paths. The disaster management solutions that will be reviewed in this work will be mobile-based only so web-based solutions that are highly cited in the literature such as Ushahidi [13] or Sahana [14] will be left to web-based service reviews. Additionally, crowdsourcing is not included if it is solely scoped to social media since there are open issues related to fake news and the reliability of such collected data [15,16]. This review aims to identify applications, systems, frameworks, or models that utilize smartphone sensors for data collection and communication, and that propose a contribution to the disaster management process leveraging crowdsensing.

There is a growing number of studies on social media or social network-based crowdsourcing in the disaster context [17,18,19], however, smartphone sensor-based mobile crowdsensing in disaster management is an understudied research area. This study contributes to the literature in explaining the current academic research and highlighting the gaps that can be investigated in future research. This paper targets both disaster management authorities (i.e., governmental administrative units, emergency response agencies) who seek guidance on the use of mobile crowdsensed data in disaster management as well as researchers who aim to contribute to mobile crowdsensing-aided disaster management solutions.

**Figure 2:**
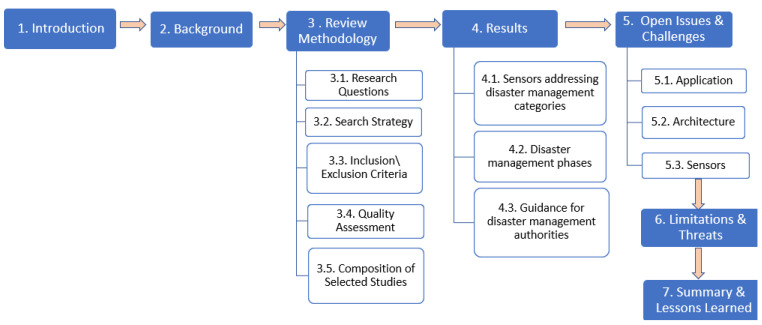
Structure of the review.

The rest of this article is structured as illustrated in Figure 2. Section 2 outlines the background focusing on the previous literature reviews conducted on the use of crowdsourcing or crowdsensing in disaster management. Section 3 outlines the detailed review methodology that is followed while conducting this systematic literature review. Section 4 presents the findings of this systematic review highlighting the answers to the research questions. Section 5 reveals the open issues and challenges, Section 6 presents the limitations and validity threats of this work and, finally, Section 7 concludes the paper with the summary findings and lessons learned.

## 2. Background

Starting from 2010, there is an increase in the number of studies on crowdsourcing in disaster management. This trend is in parallel with several disasters since 2010 such as the Haiti earthquake in 2010, US Superstorm Sandy, 2012, Colorado Wildfire, 2013, or Hawaii Hurricanes, 2014, which have contributed to channelling the research interest in finding solutions to reduce human and economic loss from such disasters. Although the literature is quite rich in crowdsourcing/crowdsensing technologies, the disaster literature utilizing crowdsourcing/crowdsensing technologies is still evolving. Few literature review studies have been noted on the use of crowdsourcing/crowdsensing in disaster management, and these reviews have been analyzed from several aspects to produce a gap analysis (Table 1) which sets the scope for this paper. A systematic literature review from 2019 [9] discusses the impact of volunteer crowdsourcing on disaster risk reduction and provides an applicability analysis of volunteer crowdsourcing studies into the disaster management cycle from the lens of geo-technology, mobile communication and digital volunteerism. This systematic review discusses the mobile communication aspect of crowdsourcing but focuses only on geo-sensors. A survey study from 2016 [20] discusses crowdsourced/volunteered geographic information but ignores the rest of the crowdsourcing tools/methods. A literature review from 2014 [21] provides a taxonomy of online tools and platforms implemented in recent years for emergency management, but focuses solely on social media and social networks. A comprehensive literature review from 2011 [22] presents a state-of-the-art review of citizen sensing in environmental and public health surveillance and crisis/disaster informatics. However, this study does not provide an analysis of crowdsensing based on smartphone sensors. Overall, existing reviews do not place emphasis on the use cases of different sensor types, build a mapping between disaster problems and sensors, or analyze the ways to integrate crowdsensing into decision support systems.

This review differentiates itself from the previous literature reviews that are summarized above in the following aspects: (1) It gives an understanding of how smartphone sensors can be used in different disaster scenarios tackling various disaster management categories or problems. (2) It focuses on relatively reliable crowd-sensed data through smartphone sensors unlike crowdsourcing through social media such as Twitinfo or Twitcident [23] or unlike web solutions that are mostly proposed in the literature such as Ushahidi [13]. (3) It builds a link between mobile crowdsensing and the disaster management cycle highlighting the phases addressed with the proposed solutions. (4) Finally, this work seeks to identify any framework or approach that is proposed by the reviewed studies to potentially guide disaster management authorities on how to integrate crowdsensed data with their decision-support systems.

**Table 1:** Gap Analysis of Previous Literature Reviews.

Publication	Year	Crowdsensing with Smartphone Sensors	Disaster Management Cycle	Disaster Management Categories	Review of Social Media Aid	Review of Decision Support Systems
Kamel Boulos et al. [22]	2011	X	X	X	√	X
Chatzimilioudis et al. [24]	2012	X	X	X	X	X
Poblet et al. [21]	2014	X	√	X	√	X
Albuquerque et al. [20]	2016	Geo-sensors only	X	X	√	X
Kankanamge et al. [9]	2019	Geo-sensors only	√	X	√	X
**This Article**	**2022**	√	√	√	√	√

## 3. Review Methodology

A systematic literature review provides a critical assessment of all research studies that address a particular research question on a research topic and unlike a standard literature review, all procedures are defined in advance to ensure transparency and replicability. This systematic literature review has been conducted in accordance with the guidelines outlined by Kitchenham et al. [25]. The research questions, the search strategy, the inclusion/exclusion criteria and the quality assessment for the selected studies are described in detail in this section. The composition of the selected studies is summarized at the end of the section.

### 3.1. Research Questions

1. How does mobile crowdsensing support disaster management through smartphone sensors?
  (a) Which smartphone sensors are used to address what types of disaster management problems?
  (b) Where do mobile crowdsensing efforts concentrate on the disaster management cycle (mitigation, preparedness, response, and recovery)?
2. What kind of guidance is proposed to the disaster management authorities by the mobile crowdsensing-aided disaster management solutions to use crowdsensed data in their decision-support systems?

### 3.2. Search Strategy

This review was primarily performed through electronic databases using keywords search. Reputable databases that provide wide coverage of engineering and science-related topics were used: IEEE Xplore, the Web of Science, Scopus, and ACM Digital Library. It is noted that Web of Science provided a larger number of studies, however, studies retrieved in ACM Digital library has higher precision.

Alternative search techniques such as subject pearl growing and snowballing are also employed to enhance the completeness and accuracy of the search. Subject pearl growing was used to improve the search query with keywords to cover the overlapping terminologies in the research topic, i.e., disaster/crisis/emergency or crowdsourcing/crowdsensing. Backward-snowballing technique is also effectively used to identify and screen the relevant studies one level deep. The studies included via backward snowballing are [14,26,27].

The search query is formulated as follows:

(crowdsens* OR crowd-sens* OR “mobile crowdsourc*” OR “mobile crowd-sourc*”) AND (disaster OR “natural hazard*” OR earthquake OR wildfire OR flood OR landslide OR hurricane OR storm OR emergency OR crisis)

The search query is run through abstract, keywords, and title search on Scopus and ACM DL, through the topic search on Web of Science, and metadata search on IEEE Xplore. The queries were run on the 11 November 2022. The search query consists of two main concepts—one for crowdsensing, one for disaster. Since the literature on mobile crowdsensing is still evolving, some terms are used interchangeably. ‘Mobile crowdsourcing’, ‘mobile crowdsensing’, or ‘crowdsensing’ all refer to the same concept of sensing through smartphones, which is the main research area of this review. Hence, mobile crowdsourcing is added to the query. ’Crowdsensing’ instead of ’Mobile crowdsensing’ is used in the query to keep the coverage of the search results broader. The terms disaster, crisis, and emergency are used interchangeably and with a significant overlap in the mainstream literature and in combinations such as crisis and emergency management or disaster crisis management [28]. Since there is a high demand for data and communication in most crisis, emergency, and disaster situations, all three terms were included in the search query to obtain more comprehensive results. Additionally, some common disaster types such as “earthquake”, “wildfire”, “hurricane”, “flood” were included in the search query in order to not miss any paper specifying the disaster type instead of using generic “disaster” or “emergency” terms. For the second research question, no separate search query was generated to identify the studies proposing guidance for disaster management authorities. Only the studies selected through the main search query were assessed to answer this question.

**Figure 3:**
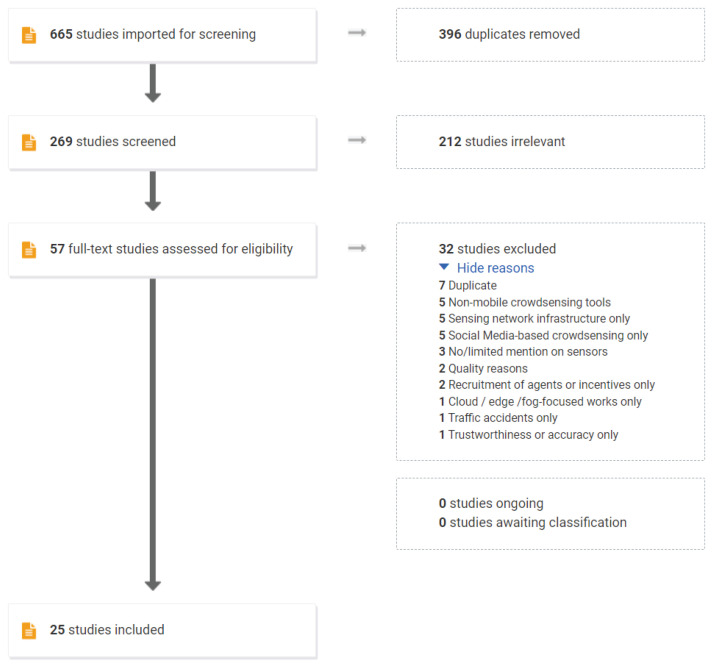
PRISMA diagram illustrating the paper selection process.

The Covidence Tool (https://www.covidence.org/) is employed during the screening and full-text review phase. The tool mainly contributed to removing duplicate studies and selection of studies in line with inclusion and exclusion criteria. The search initially produced 269 studies (after duplicates were removed), which were subsequently reduced to 56 studies eligible for full-text review. After applying quality and exclusion criteria which will be discussed in the following subsection, the final number of studies selected for this review was reduced to 25. The paper selection process is summarized in the PRISMA (http://www.prisma-statement.org/) diagram as shown in Figure 3.

### 3.3. Inclusion/Exclusion Criteria

To limit the scope of the review and stay focused on the targeted research questions, several exclusion criteria were introduced in the search process to eliminate papers focusing on:

- Recruitment of agents/incentives only;
- Non-mobile crowdsensing tools;
- Traffic accidents only;
- Trustworthiness only;
- Cloud/edge/fog-focused works only;
- Sensing network infrastructure only;
- Social media crowdsourcing only;
- No or limited mention of sensors.

### 3.4. Quality Assessment

The quality criteria listed in the bullet points below were used to ensure that good-quality papers are included in the review.

- Is the research question or objective clearly stated?
- Is there a documented research methodology?
- Are the research findings supported?
- Is the contribution of the work clearly explained?

The criteria are scored as Yes (exists), No (does not exist), or Partly (partly exists). If a paper is scored with three or more ‘No’s or four ‘Partly’s, then the paper is excluded from the review due to inadequate quality. Two studies were excluded from the review due to inadequate quality.

### 3.5. Composition of Studies

**Figure 4:**
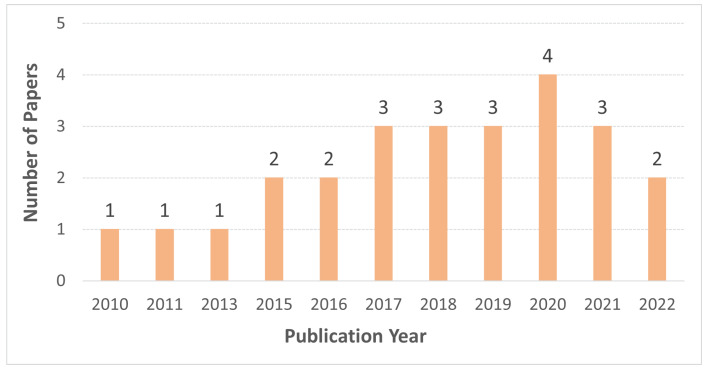
Publication Year of the Reviewed Studies.

The selected studies for this review were published between 2010 and 2022 with 80% published after 2015 (Figure 4). The reviewed studies consist of 11 conference proceedings, 10 journal articles, and 4 book chapters. From the sample, 14 studies propose conceptual solutions to disaster management, while 9 studies present empirical research results and two studies use both conceptual and empirical research designs. The evaluation methods used to assess the conceptual studies are explained in the Section 4.

## 4. Results

This work investigated three topics in the reviewed studies, each aiming to answer a research question (RQ) or a subquestion as stated below next to each subsection:

- 4.1 Use of smartphone sensors in addressing disaster management categories—RQ1.a;
- 4.2 Disaster management cycle phases targeted—RQ1.b;
- 4.3 Guidance for disaster management authorities—RQ2.

The following subsections are organized to elaborate on these topics and answer the research questions, respectively. However, it is worth highlighting some general findings of this review work before discussing the research questions.

There were no exclusion criteria set on the type of studies or the type of mobile crowdsensing-aided disaster management solutions to be included in this systematic review. The solutions proposed could be a mobile application, a system architecture or a framework. Any type of solution that employs mobile crowdsensing or makes use of smartphone sensors in the context of a disaster seeking crowd involvement is included in the scope of this work.

The majority of the studies (12 papers) propose a *System Architecture*: Frommberger and Schmid [29] present a system architecture for an integrated disaster alerting and reporting system composed of an Android app, web interface, and disaster management server named Mobile4D. Visuri et al. [30] present a two-tier system for building collapse detection in earthquakes that uses residents’ smartphones as distributed sensors. Anagnostopoulos et al. [31] propose a Four-Layer system integrating crowdsourcing, crowdsensing, and an LSTM (long short-term memory) inference model for municipality resource allocation. Bhattacharjee et al. [32] present a post-disaster map builder system including trace management and map inference modules utilizing GPS sensors in smartphones. Asiminidis et al. [33] perform an empirical study presenting a Bluetooth-based cheap and autonomous system for indoor localization determination that is tested for a fire emergency scenario in a motorway tunnel. Piscitello et al. [34] propose a system architecture for the detection and management of emergencies in smart buildings which is called Danger-System, composed of a DangerCore server structure and mobile application with two interfaces for building managers and tenants. Sadhu et al. [35] discuss the architecture for a real-time 3D mapping system of the disaster scene where data collection is made cooperative through crowdsensed data from bystander agents. Sarbajna et al. [36] introduce a decentralized mapping service relying on a blockchain backend to generate a complete, current, and accurate graph of accessible paths in a disaster-affected region to ensure any responder can contribute to the navigational map and everyone works on the last version of the map. Vahdat-Nejad et al. [37] present an information gathering system for earthquake disasters which is composed of sensing, fog, cloud, and application layers and equipped with data collection, processing, and storage capabilities. Villela et al. [13] propose a conceptual design of the RESCUER system, a smart and inter-operable decision support system that uses crowdsourced information in emergency and crisis management. Salfinger et al. [38] present a situation-adaptive system design capable of exploiting both conventionally sensed data and unstructured social media content. Gao et al. [26] discuss a conceptual system architecture of groupsourcing to facilitate efficient collaborations among various organizations responding to a disaster incident.

Four studies propose *methods*: Kitazato et al. [39] propose a method to detect real-time pedestrian flows which is believed to be crucial for disaster evacuation guidance. Li et al. [40] suggest a method to simulate the post-earthquake evacuation and mobile crowdsensing-based monitoring of citizens under instructions from a central authority. Zabota and Kobal [41] discuss a new methodology for collecting data on past rockfall events through mobile application to enhance the quality of rockfall risk assessment. Burkard et al. [42] present image-based methods for measuring the water level at small drainage areas with a mobile phone and inbuilt sensors.

Three of the reviewed studies propose a *Framework* solution: Ae Chun et al. [43] introduce a Public Engagement in Emergency Response (PEER) framework which provides an online and mobile crowdsourcing platform for incident reporting and citizens’ resource volunteering together with an intelligent recommender system to assign citizen resources with emergency tasks. A. Fahim et al. [44] discuss a data-efficient framework where only 1% of the crowd-sensed data are consumed for highly accurate detection of global events through parallelization of visual content. Kielienyu et al. [45] proposes a framework for community health monitoring to generate MCS-driven community risk mapping through collecting GPS signals from citizens’ smartphones.

Two studies propose *mobile applications*: Nguyen et al. [27] introduce a novel mobile application design for integrating crowdsourced data collection and validation activities in disaster risk reduction processes. Fajardo and Oppus [14] introduce an Android-based disaster management system, MyDisasterDroid (MDD), which determines the optimum route between volunteers and victims to serve the greatest number of people in the maximum coverage of the area.

Di Felice and Iessi [46] propose a software service to reduce the processing times of Tweets during emergencies, Ludwig et al. [23] introduce a web application using mobile crowdsensing that combines physical and digital activities to respond to rescue authorities’ information requests, Choi et al. [47] presents a cloud-based data process that employs a mobile crowdsensing application to detect images as per damage type, Tripathi and Singh [48] present a data fusion model of human virtual sensors and actual traditional sensors for disaster response.

**Figure 5:**
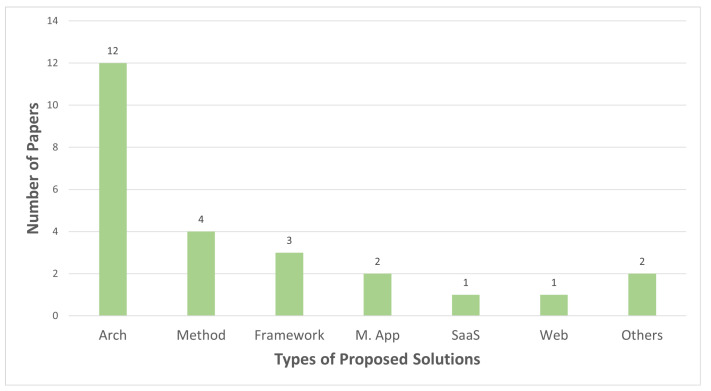
Types of MCS-aided disaster management solutions. Arch: System Architecture; M.Ap: Mobile Application; SaaS: Software as a Service; Web: Web Application.

Figure 5 summarizes the types of solutions proposed by the reviewed studies. The classification of the solutions are done in accordance with the authors’ definition of their work. The category *others* consolidates solutions such as process and model since there is one paper classified in each category.

**Figure 6:**
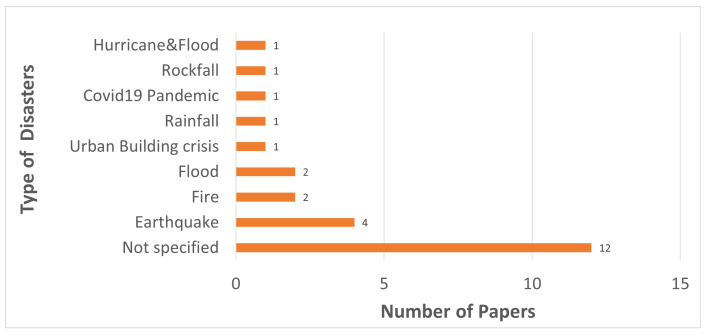
Disaster Types Targeted in the Reviewed Studies.

The **types of the disasters** (i.e., flood, earthquake, fire, hurricane) were also analyzed during the review, however, most of the studies propose generic conceptual solutions not targeted to a certain disaster type, hence, no meaningful result could be derived neither on disaster type, nor its relation to the other investigated concepts. Still, the disaster types targeted by the empirical solutions are given in Figure 6.

An important characteristic of mobile crowdsensing is whether the crowd sensing is **participatory or opportunistic**. In simple terms, participatory crowdsensing refers to the user’s active participation in sensed data generation, whereas in opportunistic crowdsensing, sensed data readings from the environment or computations through devices are performed automatically in the background [24]. The solutions are also assessed in terms of participatory or opportunistic sensing. The majority of the studies selected for this systematic literature review (16 out of 25) assume a participatory mobile crowdsensing structure where users’ active contribution was necessary. Seven studies took an opportunistic sensing approach in their proposed solutions, while two studies used both approaches. Although there is no evident reason stated to explain this situation, one can assume that the challenges in obtaining user consent for opportunistic sensing could have contributed to the smaller number of studies using opportunistic sensing.

A comprehensive list of the reviewed studies with sensors used, disaster categories addressed, disaster cycle phases targeted, or the types of solution proposed is given in Table 2.

### 4.1. Use of Smartphone Sensors in Addressing Disaster Management Categories

This section aims to answer the research question 1-a. With the advancement in sensor technologies, there is an ever-increasing number of sensors built into smartphones such as GPS, camera, microphone, accelerometer, gyroscope, barometer, pedometer, light and proximity sensors [49]. These sensors collect information on visualization (i.e., camera), localization (i.e., GPS, bluetooth), directionality (i.e., gyroscope), or mobility of objects (i.e., pedometer, accelerometer), which provide valuable information at a low cost in disaster management.

**Figure 7:**
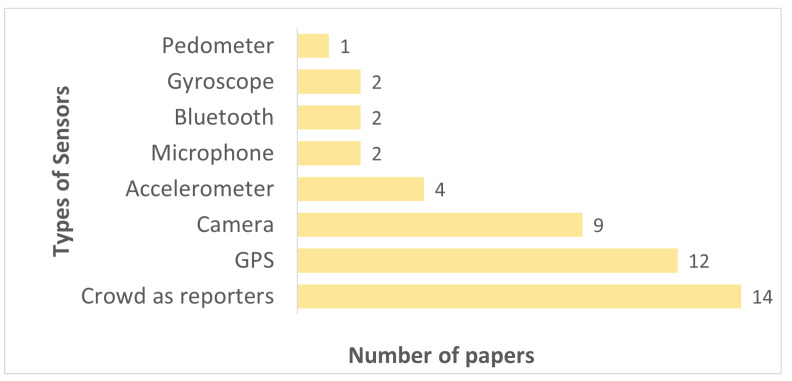
Types of Sensors Used in the Reviewed Studies.

During the review process, it was observed that the *crowd as reporters* phenomenon, where users report on a situation using the sensing capabilities of smartphones and the report gets verified by an official, appears as a frequently used sensing solution. This idea adopts the concept of smart citizens for smart cities crowdsensing [50]. Smart citizens, or *crowd as reporters* as stated in this paper, are believed to be major drivers of smart cities and they have been increasingly active in sensed data generation through smart city applications [50]. Ludwig et al. [23] define the *crowd as reporters* as users with a concise and conscious use of existing knowledge to achieve a specific task or goal. Kamel Boulos et al. [22] also refer to “human sensors” or “human-in-the-loop sensing” concepts to define the increasingly active role of humans in the sensing environment. Although this study initially intended to focus on smartphone-based sensors, after reviewing several papers that have been retrieved through the specific search query, it is concluded that human intervention still appears to be vital in disaster and emergency scenarios. Moreover, *crowd as reporters* stands as a representation of the participatory sensing of mobile crowdsensing. Hence, the *crowd as reporters* notion is recognized as a type of sensor in this review. The *crowd as reporters* sensor is used in 14 of the studies which is a considerably high number. The use frequency of the sensors in the reviewed studies is demonstrated in Figure 7.

**Figure 8:**
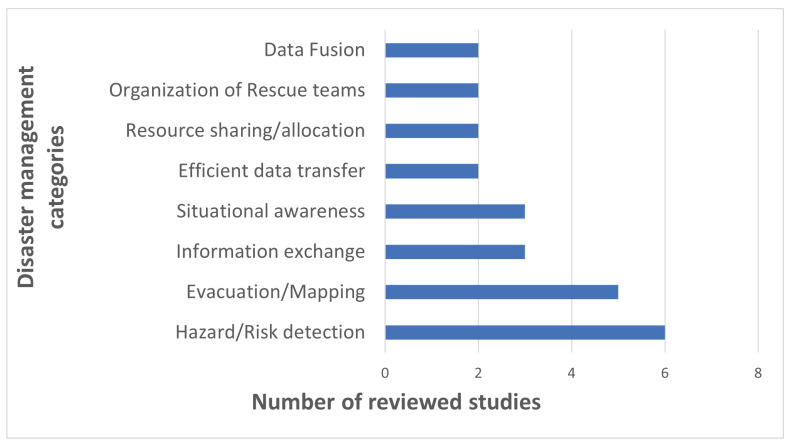
Disaster Management Categories Addressed by the Reviewed Studies.

**Figure 9:**
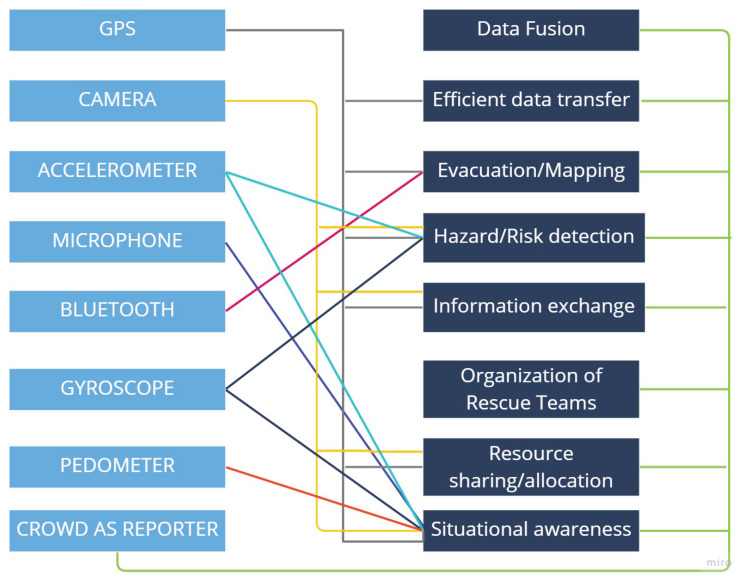
Sensors used in addressing disaster management categories.

Throughout the course of this review, disaster management problems that the reviewed studies have commonly addressed are noted and classified under eight categories such as evacuation/mapping, hazard/risk detection, organization of rescue teams, data fusion, information exchange, situational awareness, efficient data transfer, and resource sharing/allocation. The context and use of sensors in addressing each disaster management category are discussed for each paper in the remainder of this section. Figure 8 illustrates the number of reviewed papers addressing each disaster management category. The mapping of the sensor types against the disaster management categories that are addressed by each sensor type is provided in Figure 9. The results are also reported in a table format where citations are included per each sensor type and disaster management category addressed Table 3.

**Hazard/Risk detection**: Detecting the hazard or risk in the disaster-affected area is the tipping point of rescue and relief operations [51]. Six studies address this problem. Visuri et al. [30] propose a building collapse detection system that uses end-user smartphones as distributed sensors accompanied by a rule-based fall detection algorithm. It is an empirical study with an evaluation of a fall detection algorithm through lab simulations and a limited field test. The main sensor used in this solution is the accelerometer and efforts are made to detect false-positive cases. Accelerometer values from devices falling on a soft or stiff surface are measured with a higher true positive rate for drops on stiff surface (98.7% vs. 95.6% for soft surfaces). An interesting finding is that jumping with phones in hand or in pocket trigger less than 1% false positive earthquake event, while running with the phone does not trigger any false positive event. A key limitation of this study is the assumption that multiple mobile phone falls indicate a building collapse. More information on the environment should be collected through smartphone sensors to ensure that there is a building collapse. Choi et al. [47] discuss identifying major damage locations and types of the incident at the damaged site through crowdsensed image data using clustering algorithms. The study presents a cloud-based data collection, processing, and analysis process that employs a mobile crowdsensing application. This empirical study uses Icheon-si and Anseong-si rainfall 2020 data to test effective detection of incident type when image data are collected from a smartphone during an emergency. Camera and GPS sensors are used but more sensors could help to detect dynamic situations. Motivating users to share information is a challenge of this solution, however, receiving analyzed content or participating in response activities are discussed as motivating factors for users. Kielienyu et al. [45] generate MCS-driven community risk mapping to predict and prepare for future COVID-19 cases. This empirical study collects GPS signals from citizens’ smartphones and obtain their mobility patterns to estimate future movements of the detected communities and calculate a risk factor for communities ahead of time. Projected heatmaps for COVID-19 risks of the communities are good contributions to support Public Health departments’ resource allocation. However, privacy concerns and lack of incentive mechanism are shortfalls of this work. A similar study was conducted by Simsek et al. [52], providing an MCS-enabled framework for risk mapping of communities based on GPS data of smartphones and empowering autonomous vehicles to respond to the public safety needs.

Zabota and Kobal [41] discuss a new methodology to enable quick and simple on-field data collection for past rockfall events through mobile crowdsourcing. A mobile application (Collector for ArcGIS, which is part of the Esri ArcGIS platform) is designed for data collection and WEBGIS platform is used for visual Web maps. A GPS sensor is used to obtain location of past rockfalls and deposits, a camera sensor is used to obtain additional attributes such as size, dimensions, etc. The data are reported into the app by the crowd, so crowd as reporter can be noted as another sensor used in this work. Li et al. [40] discusses structural health monitoring, i.e., regional building damage assessment, through mobile crowdsensing. An earthquake strike in a city is simulated in ’Unity’ simulation environment where ‘Ground Eye’ acts as the city brain to coordinate emergency response and assign tasks to citizens to collect data on damage assessment. Acceleration is measured by the accelerometer sensor, strain, and inter-story drift are captured by camera sensor. Smartphones have to be installed on the damaged structure by the citizens to capture the time history data for all three parameters. Information exchange between citizens is overlooked in this study. Burkard et al. [42] present image-based measurement methods to feed into a conceptual flood prediction system that can be used in rivers. The methods are based on three variants of inclination, reference points, and correspondence points, which are measured using the camera and orientation sensors (accelerometer and gyroscope) of a smartphone. The methods were evaluated in a demo application for Android phones, however, the conditions were assumed to be ideal. Variant targeting correspondence points provides the most accurate prediction given that the camera image taken captures four of the previously defined reference points. The implementation of these measurement methods in a real-life setting with real users remains a big challenge since the evaluation has only been performed under ideal conditions.

**Evacuation/Mapping**: Evacuating the endangered area and guiding the victims to a safer place is one of the most important problems that need to be addressed during a disaster. In the aftermath of a disaster where the road networks are damaged and navigation through existing route networks is not possible, unconventional mapping techniques are required to mobilize the resources in the affected areas. The evacuation/mapping problem is one of the two most studied problems in the reviewed studies. Five studies discuss the evacuation/mapping problem. Three out of five studies use GPS sensor data and two studies use Bluetooth signals. Bhattacharjee et al. [32] propose a post-disaster map builder system on Android smartphones which includes trace management and map inference modules, where traces of users are collected through GPS sensors and fed into map inference module. Pedesterian maps are built by the map inference model through the collected trajectory traces. It is a conceptual study for which evaluation is performed through simulation and Testbed on ONE simulator and the Mapping Toolbox of MATLAB 2017a. The key assumption of the study is that the map builder system has to be pre-installed on all node devices. This paper pre-processes raw trajectory traces for noise reduction, performs significant point identification, and uses clustering technique and Topology inference. This study achieves successful sending of 95% of trajectory traces to their destinations in two hours of generation, which is a quite promising contribution to the mapping problem.

Asiminidis et al. [33] propose a low-cost autonomous Bluetooth Low Energy (BLE) sniffing technique to guide and show the emergency exit upon receiving the RSSI (received signal strength indication) values from users’ smartphones and thus guiding the users instantly during an emergency in a tunnel. It is an empirical study presenting a Bluetooth-based cheap and autonomous system for indoor localization determination that is tested for a fire emergency scenario in a motorway tunnel. The solution uses the Bluetooth sensor to show the emergency exit to the users using their smartphones’ RSSI values and hence poses concerns regarding user privacy. Kitazato et al. [39] discuss a system to detect real-time pedestrian flows including pedestrian congestion, direction, and velocity through mobile sensing with Bluetooth signals for evacuation route guidance during disasters. The study analyzes separately the congestion degree by detecting the surrounding Bluetooth devices and the direction and velocity of the pedestrians through the RSSI of a Bluetooth LE beacon carried by the pedestrian. Limited Bluetooth scope and battery limitation of smartphones can be a downside of this solution. Sarbajna et al. [36] present a decentralized mapping service relying on a blockchain backend, placed in a smartphone and available to everyone to generate a complete, current, and accurate graph of accessible paths in a disaster-affected region to ensure any responder can contribute to the navigational map and everyone works on the last version of the map. GPS sensor is used in this solution. The blockchain component of the solution helps with the accessibility of the system without a central authority need. It is a conceptual study with no evaluation, hence deployment in a real-world setting is needed to ensure the validity of this work. Fajardo and Oppus [14] introduce an Android-based disaster management system (MyDisasterDroid) to facilitate the logistics for the rescue and relief operations determining the optimum route between volunteers and victims to serve the greatest number of people in the maximum coverage of the area. The solution uses a genetic algorithm to determine the optimum route and receives data from GPS sensors. It is based on Google’s Android system and assumes Google Maps navigation is not distorted, but it is very likely so in a flood or earthquake disaster.

**Information exchange**: It is possible to obtain comprehensive situational information in the shortest time through mobile crowdsensing—three studies contribute to information gathering systems. Frommberger and Schmid [29] propose an integrated disaster alerting and reporting system architecture called Mobile4D for an integrated disaster alerting and reporting system composed of an Android app, web interface, and disaster management server. It is a conceptual study but the evaluation is performed through usability testing. This study tackles two important challenges: lack of institutionalization and lack of two-way communication. A top-down, bottom-up communication channel between authorities and disaster victims with an escalation procedure is designed. The system mainly uses the *crowd as reporters* sensor where users report on the situation through the system where the report gets verified by an official; it also exploits other sensory information such as GPS for location detection and a camera for sharing visual information. The solution is functional under weak network conditions and is a text-free interface to avoid misinformation, however, improvements on information visualization were noted during the evaluation phase. It was tested with a small group of people and is only limited to Android Applications. Vahdat-Nejad et al. [37] present an information gathering system architecture for earthquake disasters which is composed of sensing, fog, cloud, and application layers and equipped with data collection, processing, and storage capabilities. The system is designed as per the requirements analysis performed through interviews with Iran’s Red Crescent Society who have lived through the 2017 Kermanshah earthquake with 7.3 magnitude. GPS and camera sensors are used in the system design, however, the *crowd as reporters* sensor is also embedded through user interface to compensate the shortfall of other sensors. Each information element is tagged with GPS coordinates to produce geotagged information maps, a camera is used to gather diverse environmental information that can either be processed by machines or reviewed by humans. Processing times for the shares images and texts are left as an area for future research. Villela et al. [13] propose a conceptual design of the RESCUER system, a smart and inter-operable decision-support system that uses crowdsourced information in emergency and crisis management under the supervision of the command control body. A mobile app with a user interaction mechanism, data analysis capabilities, and views on relevant aggregated data is a part of the system. Two types of information exchange are proposed—participatory through crowd reporting and opportunistic through smartphone sensor data sharing consented to by the citizens. GPS, camera, and *crowd as reporters* sensors are used and detailed data processing steps for each type of sensed data (text, image, video) are explained by the authors.

**Situational awareness**: Situation awareness is about providing the real-time situation of the disaster area to the stakeholders, i.e., citizens, volunteers, and disaster management authorities [53]. Three studies address this problem. Piscitello et al. [34] propose a system architecture to address an urban building crisis with a proposed system called Danger-System that creates a two-way communication between residents and building administrators leveraging both building infrastructure and mobile devices to create instant situational awareness. It is a conceptual study with scenario-based evaluation and the researchers created their simulator (DangerSystem simulator) in Python 2.7. It facilitates information gathering from residents through the microphone and pedometer sensors of their smartphones to detect any dangers in a building proactively, however, privacy and limited validation of data are key concerns of this solution. Sadhu et al. [35] propose a smartphone-enabled system named Argus that generates a real-time 3D map of the disaster area including crowdsensed inputs from bystanders. Users are allowed to share data from camera, microphone, GPS, accelerometer, and gyroscope sensors of their smartphones to help with 3D reconstruction. It is a conceptual study simulating a fire scenario and evaluated through prototyping. This paper exploits the MARL framework (Multi-Agent reinforcement learning) for data collection that is implemented with a distributed Q-learning approach to direct the agents to capture data on the areas of interest. However, this framework is not evaluated in this paper. Salfinger et al. [38] discuss a situation-adaptive SAW (situation awareness) system capable of exploiting conventionally sensed data and unstructured social media data and continuously optimizing itself through situational feedback loops. It exploits the CrowdSA framework and uses Hawaii Hurricanes 2014 dataset and General Disasters (Twitter) 2014 dataset. The *crowd as reporters* sensor is mainly used in this solution. The proposed crowd-sensing enhanced SAW system architecture keeps track of the monitored real-world situation’s evolution and reuses the detected (and projected) situational context to optimize its crowd-sensing configuration and processing. Situational feedback loops are introduced in between processing levels to improve situational awareness. The system is tested on limited datasets so large-scale studies on various types of crises would be necessary. It is noted that to gain situational awareness of the disaster area, social media data and human input are still heavily consulted.

**Efficient data transfer**: During a disaster or a crisis, an extensive amount of information is shared either on social media or on conventional emergency platforms. However, it is highly critical to transfer data effectively and efficiently during a disaster, considering the limited bandwidth and intermittent connectivity [54]. Honarparvar et al. [55] address the energy consumption problem in wireless sensor networks where sensor nodes are located far from the base stations. The authors propose an integrated location based social network that can reduce energy consumption up to 42% through reduced routes to BSs. In our review, there are two studies that discuss efficient data transfer issue. Fahim et al. [44] present a novel data-efficient framework to transfer very limited data to the central server and yet still detect global events or disasters with high accuracy. Only 1% of the crowd-sensed data are consumed for highly accurate detection of global events. Through parallelization of visual content, the average delay of content retrieval is reduced by 67%. This solution can help use the limited bandwidth and connectivity more efficiently and effectively during a disaster. Felice and Iessi [46] introduce a software service to reduce processing times of Tweets during emergencies to speed up the analysis of post-earthquake on-site messages. Efficient data transfer in limited bandwidth and limited battery life is a key problem in mobile crowdsensing during disasters and it is a topic to be further explored to help increase the utilization of smartphones in emergencies. This empirical case study uses GPS and the *crowd as reporters* sensors through the TwittEarth mobile app. Territorial data of Abruzzo region (center of Italy), an area repeatedly affected by destructive earthquakes, as provided by the Italian Institute of Statistics, and OpenStreetMap Data are used for this study. The study presents promising results in terms of fast data processing to identify exact damaged locations, however, the test sample was not very inclusive since a low number of Twitter users turn on location sensors to preserve privacy.

**Resource sharing/allocation**: Sharing and allocating scarce resources during a disaster is a major problem and is one of the key reasons for communicating in disasters [56]. Two studies discuss this problem. Ae Chun et al. [43] introduce the PEER Framework with a vision of a centralized database to facilitate resource sharing capabilities and an intelligent recommender system to match citizen resources with emergency tasks. No specific sensor type was mentioned except for the *crowd as reporters*. The conceptual framework aims to support a comprehensive and unified disaster management system integrating social media channels and smartphones. It is evaluated through a Flood Warning Community System prototype, however, the prototype is not tested. Anagnostopoulos et al. [31] try to solve the resource allocation problem of municipalities and propose a resource allocation system that can potentially be used for efficient disaster planning. The study uses a Greece Papagos–Cholargos municipality dataset (a smart city located in Athens, Greece) and utilizes Citify Crowdsourcing System Architecture as a data processing platform. The proposed system is composed of four layers. The first layer is the environmental crowdsourcing—the physical layer of the municipality where citizens are located and perform crowdsourcing activities. The second layer is the smartphone crowdsensing where citizens act as human sensors and annotate the environmental situation with the use of sensors. The third layer is the inference engine model which makes resource allocation (assigns problems to specific departments) possible and with the help of the LSTM classifier, the system can propose solutions for future cases. In the last layer, municipality headquarters’ staff work on the solution to the disaster recovery problem. The proposed system heavily relies on crowd reporters as a sensor. Spatial positional data, camera or audio data obtained through smartphone sensors are not further used by the system but instead they are used for annotation. Although this study describes a comprehensive system architecture, the evaluation is largely performed on the inference model and prediction accuracy. It could be an interesting topic to analyze autonomous resource allocation among distributed parties.

**Organization of Rescue teams**: Coordinating private rescue and relief activities during disasters became very common with the rise of social media [57]. Two studies try to propose a more structured method for organizing rescue teams. Ludwig et al. [23] introduce a web application called CrowdMonitor using mobile crowdsensing principles to align official emergency services with physical and online volunteer activities. The main objective of the study is to examine the potential of social media-generated information for situation assessment and at the same time, the potential for involving volunteers into the current work of emergency services avoiding duplications or conflicts in responding to authorities’ information requests. The crowd as reporter is the main sensor used in this study to report the requested information either on Open Street Map or in the mobile application. Gao et al. [26] discuss an approach to facilitate efficient collaborations among various organizations responding to a disaster incident. This solution highlights a gap in other crowdsensing applications which is the lack of a central authority and a unified mechanism. Hence, the study suggests a central authority that will ensure data integrity and quality, and a subscription mechanism for other response groups which is named as “groupsourcing”.

Organizing rescue teams need a more structured approach than casual efforts on social media in order to prevent duplicate efforts, speed up responses and ensure reliability of shared data. The reviewed studies have taken a conceptual and qualitative point of view, without utilization of sensor data other than the *crowd as reporters*.

**Data Fusion**: Data fusion refers to the integration of heterogeneously sensed data from actual traditional sensors with the crowdsourced data to provide a more comprehensive understanding and an improved information structure [58]. Two studies reviewed in this work discuss elements of data fusion in responding to disasters. Tripathi and Singh [48] introduce C-Sense as a new paradigm of heterogeneous crowd sourcing and investigate the impact of training on volunteer participants’ contribution to disaster operations. The study presents a data fusion model of human virtual sensors and actual traditional sensors for disaster response. The crowd as reporter sensor is heavily used in this solution. Random and trained participants are evaluated for their contribution in the participatory sensing. This model envisions real-time data collection from various communication sources but the complexity of sensors appears as a challenge. Nguyen et al. [27] introduce a novel mobile application design for integrating crowdsourced data collection and validation activities in disaster risk reduction processes. Heterogeneous data from ‘crowd as reports’ sensors as disaster reports or communication through notifications and related social media posts are integrated in the design. The key contribution is adding a validation mechanism for others’ reports by up-voting or down-voting to mitigate unreliable data from social media. The reviewed studies emphasize the significance of data quality and reliability in data fusion which is also one of the key problems of crowdsourcing. The value of data is a quite critical concept in prioritizing the data and hence enhancing the quality of fused data.

### 4.2. Disaster Management Phases Targeted

This section aims to answer research question 1-b. Disaster management is an integrative process consisting of mitigation and preparedness (before a disaster), response (during a disaster), and recovery (after a disaster) phases [59]. The mitigation phase refers to activities to prevent or reduce the potential damage through developing regulations, conducting a risk analysis, buying insurance, or organizing informative training on mitigation strategies. The preparedness phase refers to activities that are organized when a disaster is likely expected [60]. Activities that contribute to saving lives, preparing for response and rescue operations, stocking for required supplies (i.e., food, water) or planning for evacuations can be listed as preparedness activities. The response phase stands for taking action like evacuating, rescuing, providing shelter and humanitarian assistance [61]. Finally, recovery phase refers to repair and reconstruction efforts to go back to normal [62].

The reviewed studies are analyzed for their contribution to the disaster management cycle. There may be studies addressing more than one phase such as response and recovery or preparedness and response. One study may have been counted more than once, hence, the total of studies stated in this section are more than the count of the reviewed papers. The vast majority of the reviewed work is targeting response activities (21 studies). Recovery is the second most studied phase in the disaster management cycle (8 studies), followed by preparedness phase (6 studies). Only two studies have concentrated on mitigation efforts. Due to the nature of mobile crowdsensing, it is normal to utilize this technology when the need for real-time data is high, hence, response and recovery efforts are mostly addressed by the reviewed studies. Moreover, additional effort is required for data sense-making or for applying predictive models, i.e., machine learning algorithms on the sensed data to predict and mitigate the risks. Hence, there is not much research that features an interdisciplinary effort to mitigate the risk of economic or human loss in a disaster.

**Figure 10:**
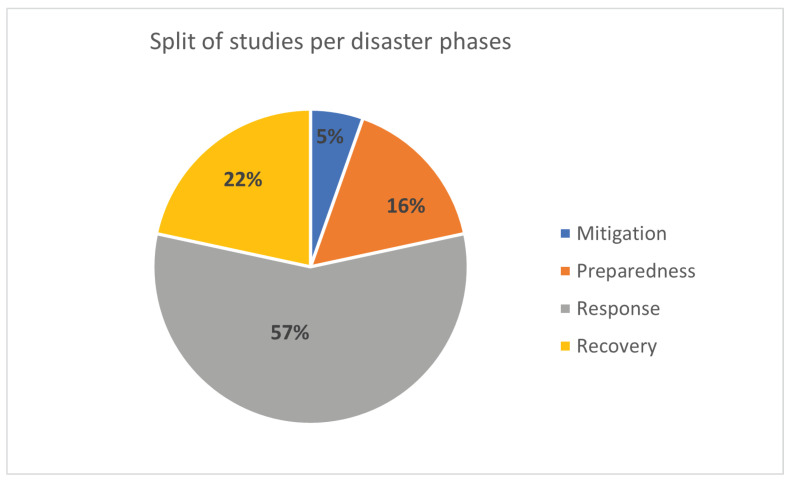
Split of studies per targeted disaster cycle phase.

Figure 10 shows the split of the studies as per the disaster management phases they target. More detailed presentation of the mapping of each study to the related phase is shown in Table 2.

### 4.3. Guidance for Disaster Management Authorities

This section aims to answer the second research question (RQ2), which seeks to identify any guidance in the form of a framework, approach, or business model that is proposed in the reviewed studies to guide the disaster management authorities or organizations through the use of crowd sensed data in their decision-support systems. Out of 25 reviewed studies, only 4 studies are deemed to propose a process-based guidance for disaster management authorities to handle crowdsensed data in their operations.

There are existing studies proposing business models or frameworks on decision support for mobile crowdsensing for participant recruitment [63], incentive mechanisms [64], task allocation [65,66], optimum sensing coverage [67], or task execution under budget constraints [68]. However, what is sought in this study is more of a holistic and process-based guidance for disaster management authorities helping them either to select the MCS-based disaster management solution that can address their needs, or to integrate the proposed solution into their existing decision-support systems.

Four criteria have been defined to determine whether a study also proposes guidance on how to integrate the solution in decision making while explaining the technicality of the solution:

- *Existence of a data flow process*: Are the data generation, data collection, data processing, or data storage processes explained? Are there references on the server technology, communication infrastructure, data collection or generation platform, bandwidth requirements or data quality measures to let the authorities make realistic choices?
- *Definition of an information exchange mechanism*: How does the information dissemination take place? Is there a two-way communication mechanism built in the solution between volunteers and disaster authorities or are there subordinate units?
- *Incorporation of human factors*: Is human behaviour modelling or user feedback taken into consideration in the design or improvement of the solution?
- *Evaluation of the proposed solution*: Is there any type of evaluation performed for the solution such as prototyping, simulation, or field testing?

The selected studies are reviewed for those criteria and only the ones that meet all four criteria are deemed fit to propose guidance to disaster authorities. Four studies are concluded to encompass the existence of a data process flow, an information exchange mechanism, the incorporation of human factors to design or fine-tune the solution, as well as the evaluation of the solution. The assessment results of the identified studies based on these criteria are summarized in Table 4.

The majority of the reviewed studies focus on the data collection, generation, or processing steps of a specific solution. However, a holistic view of the full process from data generation to decision making is critical to speed up the adoption of mobile crowdsensing in disaster management.

It should be noted that no separate search query has been generated to identify studies proposing guidance for disaster management authorities. Most probably, there are other studies driven to provide guidance on the integration of crowdsensed data in current disaster management decision support systems. However, for this review work, only the studies selected through the main search query have been assessed for the existence of process-based guidance on the integration of crowdsensed data.

## 5. Open Issues and Challenges

### 5.1. Application

The testing of disaster management solutions in real-life settings is a key challenge for researchers due to cost concerns and the lack of disaster simulation environments. Hence, the majority of the reviewed studies are conducted at a conceptual level.

With regards to the analysis of the studies for their contribution to the disaster management cycle, it stands out that the mitigation phase of the disaster management cycle is the least studied. This can be due to the nature of mobile crowdsensing which provides real-time data and hence is preferred mostly in the planning of response and recovery actions. However, still some mitigating actions such as detecting high-risk areas, developing policies and regulations, studying human factors with a user-centric approach, and training for the citizens and/or volunteers can be designed with the help of mobile crowdsensing. This can be an area of interest for future research.

This review reveals that there is not much guidance in the literature for disaster management authorities to integrate crowdsensed data into their decision-support systems, except for some conceptual frameworks proposed. Considering the recency of this technology and the potential contribution it can make to saving human lives and limiting economic losses in a disaster incident, more research, preferably interdisciplinary research, is required to make this technology more approachable as well as more applicable to existing decision-support systems. Developments are being made in silos, but when they are combined in an interdisciplinary effort, each dimension may need to concede a less optimal solution.

Technology innovation is not at the same pace as regulation, posing a challenge to the application of technology-based solutions. Finally, varying conditions across jurisdictions to use data is another challenge in data-oriented technologies. A regulatory framework is also crucial in the adoption of crowdsensing technologies [69], but there is no mention of the legal aspect or privacy concerns on data usage in the reviewed studies.

### 5.2. Architecture

A key and common challenge of the solutions is that they are dependent on the connectivity of the network which is likely to be damaged in a disaster incident. Efficient data transfer with limited bandwidth and limited battery life are also other critical weaknesses in mobile crowdsensing. Massive IoT implementation under 5G architecture can facilitate MCS-based solutions [70], but this requires ultra-reliable low-latency communication (URLLC) and assessment of data veracity in real time over URLLC links [71]. The overview of the existing crowdsensing solutions in disaster situations has also presented some ideas on the open issues in mobile crowdsensing architecture in general. The solutions reviewed for this work mostly focus on the data collection process and underestimate the data processing, storage, and dissemination processes. Additionally, data validation activities are not discussed in sufficient detail for crowdsensed data. Data validation is concerned with truth discovery in MCS [72], and most of the reviewed systems adopt a collect-and-report model, whereby collected data are directly pushed onto the networking infrastructure without assessing the quality or value of the data. As mobile crowdsensing becomes more widespread, it is likely that more research effort will be channelled to assessing the quality as well as value of the collected data.

### 5.3. Sensors

Although there is a growing number of studies on the use of crowdsourced data in disaster management, the literature on smartphone sensor-based crowdsensing in disaster management is still in its infancy. GPS and camera sensors stand out as the most utilized sensors among all the available sensors in smartphones. However, more information on the environment should be collected through various smartphone sensors simultaneously to be prepared for, to respond to, or to recover from a natural disaster or hazard. The scarcity of physical sensors is a barrier for tapping the full potential of mobile crowdsensing. Hence, heterogeneous crowdsensing, meaning the harmonious integration of virtual human sensors with the conventional sensors, is a rising research area that aims to meet the instantaneous data need for improved decision making in disaster management.

## 6. Limitations and Threats

The types of threats and limitations that are discussed in this section were inspired by Perry et al. [73]. **Threats:** Overlapping concepts in the research topic (crowdsourcing vs. crowdsensing or emergency/disaster/crisis) may adversely impact the construct validity of this review. Additionally, there might be some relevant studies that are were not found or included in the review. To mitigate this risk, the full-text review of the studies is supported by alternative search techniques and the thesis supervisor of the author as well as the course instructor were consulted. External validity, in other terms, generalizability, is a key concern for this review because most of the studies are conceptual. This risk could not be fully mitigated, however, the evaluation technique used in each reviewed solution is stated to inform the readers about this risk.

**Limitations**: Having one researcher dedicated to this review might impose some risk on the internal validity of this review. Including only peer-reviewed studies in this review supports the construct validity, however, including only academic perspective might not reflect current industry practices. Additionally, to minimize researcher bias, other authors and peers were consulted for feedback.

Despite these limitations and threats, the research results shed light on the current research on the use of mobile crowdsensing in disaster management.

## 7. Summary and Lessons Learned

This paper has presented a systematic review of the use of mobile crowdsensing technology in disaster management. Twenty-five peer-reviewed studies, mainly selected through automated database search, were reviewed and discussed. Emerging crowdsensing technology can provide real-time and reliable information to support the decision making of disaster management authorities. Despite the growing number of studies on social media-based crowdsourcing in disaster management, smartphone sensor-based crowdsensing in disaster incidents appears as an understudied research area. Only a few of the smartphone sensors are continuously being utilized (i.e., GPS, camera) and human input is still heavily required in the data collection process (i.e., crowd as reporters). This paper contributes to the disaster literature by mapping the smartphone sensors to the key disaster management categories that are produced for this work, according to the potential use cases of sensors in disaster incidents.

Another contribution of this review paper is to highlight which phases of the disaster management cycle the mobile crowdsensing efforts are currently targeting and where else they can focus. It seems that the mitigation phase is the most understudied area, mostly due to the need to integrate other technologies such as predictive machine learning algorithms into the solution to propose mitigating actions. The response phase is the most commonly studied area since the demand for real-time data is the highest. One of the key takeaways of this systematic literature review is that the testing of conceptual disaster management solutions in real-life settings is a key challenge for researchers.

Finally, four criteria have been defined to conclude if a study provides a potential guidance to authorities in implementing crowdsensing technology in their decision support systems: existence of a data flow process, definition of an information exchange mechanism, incorporation of human factors, and evaluation of the proposed solution. Four of the reviewed studies met the expected criteria and are listed in Section 4.3 for future reference of interested disaster authorities or scholars.

## Figures and Tables

**Table 2:** List of the Reviewed Studies.

				Phases in Disaster Management Cycle			
**Publication**	**Proposed Solution**	**Disaster Management Categories Addressed**	**Sensors Used**	**Mitigation**	**Preparedness**	**Response**	**Recovery**
Bhattacharjee et al. [32]	System architecture	Evacuation/Mapping	GPS				X
Asiminidis et al. [33]			Bluetooth			X	
Sarbajna et al. [36]			GPS			X	X
Frommberger and Schmid [29]		Information exchange	GPS, camera, crowd as reporters		X	X	X
Vahdat-Nejad et al. [37]			GPS, camera, crowd as reporters			X	
Villela et al. [13]			GPS, camera, crowd as reporters		X	X	
Piscitello et al. [34]		Situational awareness	Microphone, pedometer		X	X	X
Sadhu et al. [35]			Camera, microphone, gyroscope accelerometer, GPS			X	
Salfinger et al. [38]			Crowd as reporters			X	X
Anagnostopoulos et al. [31]		Resource sharing/allocation	Crowd as reporters		X	X	
Visuri et al. [30]		Hazard/Risk detection	Accelerometer			X	
Gao et al. [26]		Organization of rescue teams	Crowd as reporters			X	
Ae Chun et al. [43]	Framework	Resource sharing/allocation	GPS, camera, crowd as reporters			X	
Fahim et al. [44]		Efficient data transfer	Crowd as reporters			X	
Kielienyu et al. [45]		Hazard/Risk detection	GPS		X		
Kitazato et al. [39]	Method	Evacuation/Mapping	Bluetooth			X	
Zabota and Kobal [41]		Hazard/Risk Detection	GPS, camera, crowd as reporters	X			
Li et al. [40]		Hazard/Risk Detection	Accelerometer, camera			X	X
Burkard et al. [42]		Hazard/Risk Detection	Accelerometer, gyroscope, camera	X			
Nguyen et al. [27]	Mobile application	Data Fusion	Crowd as reporters		X	X	X
Fajardo and Oppus [14]		Evacuation/Mapping	GPS, crowd as reporters			X	
Di Felice and Iessi [46]	Software service	Efficient data transfer	GPS, crowd as reporters			X	X
Ludwig et al. [23]	Web application	Organization of rescue teams	Crowd as reporters			X	
Choi et al. [47]	Process	Hazard/Risk detection	Camera, GPS			X	X
Tripathi and Singh [48]	Model	Data Fusion	Crowd as reporters			X	
			**Total**	**2**	**6**	**21**	**8**

**Table 3:** Citations for sensors used in addressing disaster management categories.

	Data Fusion	Efficient Data Transfer	Evacuation/Mapping	Hazard/Risk Detection	Information Exchange	Organization of Rescue Teams	Resource Sharing/ Allocation	Situational Awareness
**GPS**	X	[46]	[14,32,36]	[41,45,47]	[13,29,37]	X	[43]	[35]
**Camera**	X	X	X	[40,41,42,47]	[13,29,37]	X	[43]	[35]
**Crowd as reporter**	[27,48]	[44,46]	[14]	[41]	[13,29,37]	[23,26]	[31,43]	[38]
**Accelerometer**	X	X	X	[30,40,42]	X	X	X	[35]
**Microphone**	X	X	X	X	X	X	X	[34,35]
**Bluetooth**	X	X	[33,39]	X	X	X	X	X
**Gyroscope**	X	X	X	[42]	X	X	X	[35]
**Pedometer**	X	X	X	X	X	X	X	[34]

**Table 4:** Studies proposing process-based guidance.

Reference	Contribution of the Paper	Data Flow Process	Information Exchange	Human Factors Integration	Evaluation
Frommberger and Schmid [29]	System Architecture: Mobile4D Crowdsourced Disaster System	Yes	Yes	Yes	Yes
Ae Chun et al. [43]	Framework: PEER Citizen-to-Citizen Resource Sharing in Emergency	Yes	Yes	Yes	Yes
Bhattacharjee et al. [32]	System Architecture: Post-disaster digital pedestrian map builder	Yes	Yes	Yes	Yes
Vahdat-Nejad et al. [37]	System Architecture: Information Gathering of Earthquake Disasters	Yes	Yes	Yes	Yes

## Data Availability

Not Applicable.
